# Immune Checkpoint and Other Receptor-Ligand Pairs Modulating Macrophages in Cancer: Present and Prospects

**DOI:** 10.3390/cancers14235963

**Published:** 2022-12-02

**Authors:** Yuanyuan Yang, Weijie Zhang, Peixiang Lan

**Affiliations:** 1 Institute of Organ Transplantation, Tongji Hospital, Tongji Medical College, Huazhong University of Science and Technology, Wuhan 430030, China; 2 Key Laboratory of Organ Transplantation, Ministry of Education, NHC Key Laboratory of Organ Transplantation, Key Laboratory of Organ Transplantation, Chinese Academy of Medical Sciences, Wuhan 430030, China

**Keywords:** tumor-associated macrophages, tumor microenvironment, immune checkpoint blockers, immune checkpoint inhibitors, cancer, immune therapy, anti-tumor immunity, immunosuppression, myeloid-targeted therapies

## Abstract

**Simple Summary:**

Macrophages exert a dual role in tumor progression. Macrophage-centered immunotherapies, including immune checkpoints (ICs) stimulation and inhibition, have demonstrated promising clinical perspectives. However, due to immune or metastatic heterogeneity, different cancer patients respond differently to these immunotherapies. Therefore, it is necessary for researchers to further discover and understand novel ICs on macrophages in the tumor microenvironment to design personalized immunomodulatory therapies to overcome therapy resistance. Based on this, we more comprehensively summarize a large number of IC and other receptor-ligands between macrophages and tumor cells, as well as their biology and role in anti-cancer immunity.

**Abstract:**

Immunotherapy, especially immune checkpoint blocking, has become the primary anti-tumor treatment in recent years. However, the current immune checkpoint inhibitor (ICI) therapy is far from satisfactory. Macrophages are a key component of anti-tumor immunity as they are a common immune cell subset in tumor tissues and act as a link between innate and adaptive immunity. Hence, understanding the regulation of macrophage activation in tumor tissues by receptor-ligand interaction will provide promising macrophage-targeting strategies to complement current adaptive immunity-based immunotherapy and traditional anti-tumor treatment. This review aims to offer a systematic summary of the current advances in number, structure, expression, biological function, and interplay of immune checkpoint and other receptor-ligand between macrophages and tumor cells.

## 1. Introduction

Cancer cells evade immune surveillance to avoid recognition and elimination by the immune system. A significant mechanism is through the immune checkpoint (IC) pathways. Immune checkpoints are comprised of stimulatory and inhibitory receptors that bind to their natural ligands and can control the type, intensity, and period of the immune response to maintain physiological homeostasis. However, cancer cells can dampen the immune response through over-expressing inhibitory immune checkpoint molecules [1]. Based on this immune evasion mechanism, immunotherapy, which activates stimulatory immune checkpoints or blocks inhibitory immune checkpoints to boost anti-tumor immune responses, serves as a supplement to tumor therapy after surgery, radiotherapy, and chemotherapy [2].

In the beginning, researchers paid more attention to investigating the adaptive immune system, especially T cells, such as Programmed Cell Death 1/Programmed Cell Death Ligand 1 (PD-1/PD-L1), Cytotoxic T-Lymphocyte Associated Protein 4 (CTLA-4) [3]. To date, 16 different immunomodulators have been approved by the FDA for more than 12 types of cancer [4] (Table 1). In addition, clinical concerns have been identified that only a minority of patients would benefit from IC therapy [5], most possibly due to immune or metastatic heterogeneity [6]. Thus, to identify and target novel immune checkpoint receptor-ligand pathways with better response rates, other components of the tumor microenvironment (TME) have been further investigated.

**Figure 1:**
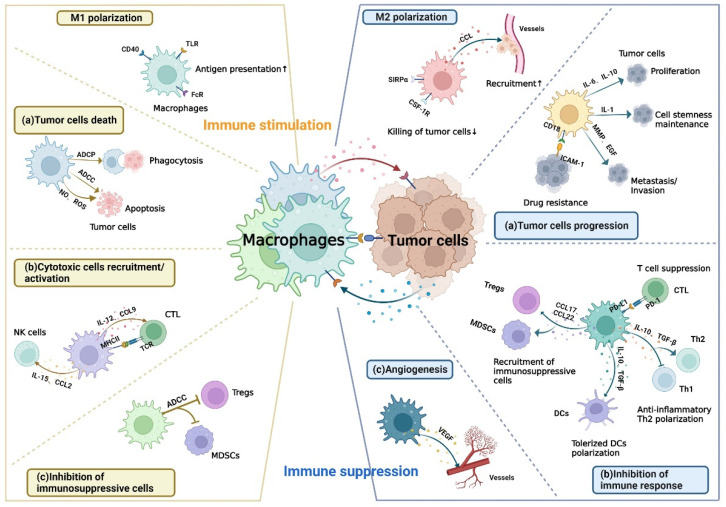
The dual role of macrophages in TME. Left: immune stimulation. Signals from tumor cells can induce M1 polarization with the increase in antigen-presenting capacity. M1 macrophages exert anti-tumor effects in three ways include (**a**) phagocytosing tumor cells and triggering apoptosis of them; (**b**) secreting cytokines to recruit and activate CTL or NK cells and presenting tumor antigens to CTL; (**c**) inhibiting immunosuppressive cells, including Tregs and MDSCs. Right: immune suppression. Signals from tumor cells can also induce M2 polarization with the decrease in the killing capacity of tumor cells. M2 macrophages can produce chemokines to recruit macrophages to TME and contribute to tumor immune escape in three ways include (**a**) promoting tumor proliferation, metastasis, invasion as well as drug resistance and maintaining tumor cell stemness; (**b**) inhibiting pro-inflammatory response by suppressing CTL, inducing the polarization of anti-inflammatory Th2 and tolerized DCs, and recruiting immunosuppressive cells; (**c**) enhancing angiogenesis to promote tumor progression. ADCC, antibody-dependent cellular cytotoxicity; ADCP, antibody-dependent cellular phagocytosis; CCL, C–C motif chemokine ligand; CTL, cytotoxic T lymphocytes; CSF, colony-stimulating factor; DCs, dendritic cells; EGF, epidermal growth factor; FcR, Fc receptor; ICAM, intercellular adhesion molecule; IL, interleukins; MDSCs, myeloid-derived suppressor cells; MHC, major histocompatibility complex; MMP, matrix metalloproteinase; NO, nitric oxide; NK, natural killer; PD, programmed cell death; ROS, reactive oxygen species; SIRPα, signal regulatory protein α; TGF, transforming growth factor; Th, helper T cell; TLR, toll-like receptor; Tregs, regulatory T cells; VEGF, vascular endothelial growth factor.

Tumor-associated macrophages (TAMs), a crucial component of anti-tumor innate immunity, are among the most abundant population in TME [7]. The capability of macrophages is significant for bridging innate and adaptive immunity, such as phagocytosis of pathogens and damaged cells, presentation of antigens, and secretion of cytokines [8]. TAMs are classified into two major subpopulations: classically activated macrophages (M1) and alternatively activated macrophages (M2). In contrast to M1, which have anti-tumor effects, M2 are involved to some extent in tumor progression and metastasis [9] (Figure 1). Several immune checkpoints have been shown to be dysregulated in TAMs and promote cancer evasion. For example, CD47–signal regulatory protein α (SIRPα) axis has been proven critical for the tumor to escape from macrophage-mediated phagocytosis [10]. Additional IC may be identified and therapeutically targeted to provide effective and individualized immunomodulatory therapies to eradicate tumor cells. In this review, we will provide a comprehensive summary of the current advances in the number, structure, expression, biological function, and interaction of immune checkpoint and other receptor-ligand between macrophages and tumor cells, which may lead to the development of new approaches to combating macrophage-associated therapeutic resistance.

## 2. Immune Checkpoint Receptor-Ligand Interaction Dependent on Cell-Cell Contact

**Figure 2:**
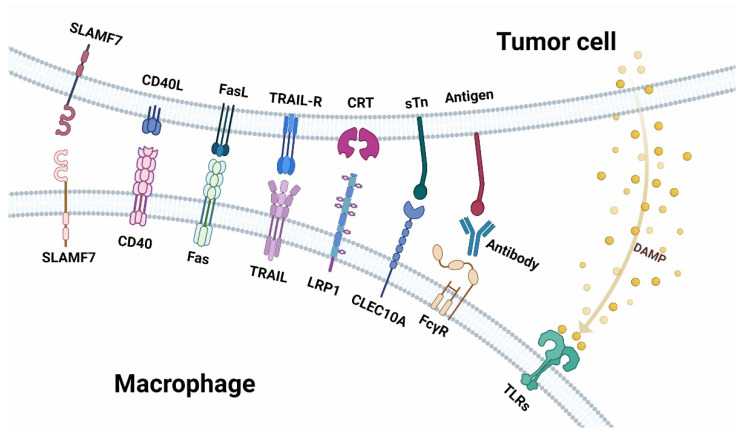
Receptor-ligand interaction between macrophages and tumor cells in anti-tumor immunity. Top: the ligands expressed on tumor cells and the molecules secreted by tumor cells. Below: are the receptors expressed on macrophages.

**Figure 3:**
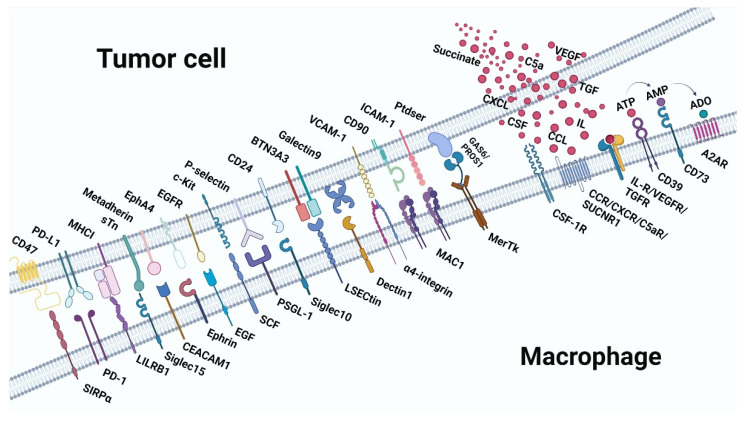
Receptor-ligand interaction between macrophages and tumor cells in pro-tumor immunity. Top: the ligands expressed on tumor cells and the molecules secreted by tumor cells. Below: are the receptors expressed on macrophages. A2AR, adenosine 2A receptor; ADO, adenosine; AMP, adenosine monophosphate; ATP, adenosine triphosphate.

Macrophages have a dual role in TME. On the one hand, as innate immunocytes, macrophages recognize malignant cells and clear them by phagocytosis, recruitment and activation of other immune cells, and secretion of pro-inflammation cytokines (Figure 2). On the other hand, researchers have found that macrophages can also contribute to cancer progression and therapeutic resistance (Figure 3). In this section, we will discuss the ligand-receptor pairs that are dependent on tumor-macrophages contacts.

### 2.1. Rreceptor-Ligand Interaction in Anti-Tumor Immunity

#### 2.1.1. FcγR-Antibody-Antigen Axis

Fcγ receptor (FcγR) belongs to a family of cell surface receptors that bind to the Fc domain of immunoglobulins and is expressed in various hematopoietic cells, including macrophages, B cells, and monocytes. FcγR consists of six members (FcγRI, FcγRIIA, FcγRIIB, FcγRIIC, FcγRIIIA, and FcγRIIIB) [11], of which most are phagocytosis-activating receptors with the immunoreceptor tyrosine-based activation motif (ITAM), except FcγRIIB, which is the only phagocytosis-inhibitory receptor with the immunoreceptor tyrosine-based inhibitory motif (ITIM) [12]. FcγRs can identify antigens in tumors through binding to antibodies and play a key role in killing or phagocytosis of tumor cells via antibody-dependent cellular cytotoxicity (ADCC) or antibody-dependent cellular phagocytosis (ADCP), respectively [13]. Interestingly, although M2 are traditionally considered pro-tumor, it may exert stronger phagocytosis than M1 to play an anti-tumor role mediated by antibodies such as rituximab in the lymphoma microenvironment [14,15].

When bound to antibodies, the FcγR domain on macrophages activates downstream signals. Accordingly, the tyrosine residues in the ITAM will be phosphorylated by SRC family kinases, which results in the recruitment and activation of the spleen tyrosine kinase (SYK) family, which transduces downstream signals that eventually lead to actin cytoskeleton rearrangement, the formation of phagocytic synapses between macrophages and tumor cells, and phagocytosis of target cells [12]. Furthermore, monoclonal antibody-based strategies have shown prominent effects in eliminating cancer cells, such as acute lymphocytic leukemia [16], ovarian cancer [17], and breast cancer [18]. In addition, FcγR-deficient mice that lacked the FcγRI and FcγRIII exhibited weaker functions of macrophages in arresting and engulfing cancer cells. On the other hand, attenuated binding of antibodies to FcγRIIB demonstrated elevated phagocytosis of cancer cells in lymphoma and leukemia mouse models [16]. Moreover, Morrissey et al. designed a family of chimeric antigen receptors with CAR structures for phagocytosis (CAR-P), which can trigger enhanced engulfment of macrophages to tumor cells [19].

#### 2.1.2. CD40-CD40L

CD40 (also known as Bp50 and TNFRSF5), a cell surface type I costimulatory glycoprotein, contains an extracellular domain, a cytoplasmic tail, and a transmembrane domain belonging to the tumor necrosis factor (TNF) family. CD40 is ubiquitously expressed in hematopoietic and nonhematopoietic cells, especially B cells and myeloid cell lineage [20]. CD40L (also known as gp39, CD154, and TNFSF5), identified as the natural ligand of CD40, is a 50-kDa type II membrane glycoprotein that is also broadly expressed in various cells [20], including tumor cells [21]. The CD40/CD40L axis actives not only the RAFs and NF-κB signaling but also the Janus kinase 3 (JAK3) and signal transducer and activator of transcription (STAT) pathway. It is involved in the maturation, survival, and cytokine production of antigen-presenting cells (APCs) to lead to the activation of T cells [22].

CD40 agonists have been proven effective in stimulating TAMs to degrade fibrosis required for tumors and elicit effective T-cell responses, thereby forming a pro-inflammatory environment [23]. In contrast to agonist therapy, increasing the CD40/CD40L interaction between tumor cells and macrophages is also a promising treatment for cancer. For example, Eriksson et al. demonstrated that after the transfer of trimerized membrane-bound extracellular CD40L (TMZ-CD40L) in pancreatic cancer cells, the TMZ-CD40L-expressing tumor cells could enhance the infiltration of T cells and M1 macrophages in tumors, ultimately inducing anti-tumor immunity [24]. A similar effect has been verified in murine lung cancer [25] and bladder cancer models [26].

#### 2.1.3. Fas-FasL

Fas (also known as CD95 or APO-1) is a 45-kDa type I transmembrane protein that belongs to the TNF receptor superfamily. Widely present in cells or tissues, Fas is made up of a transmembrane domain, an intracellular death domain (DD), and an N-terminal domain containing three cysteine-rich domains (CRDs) [27]. FasL, the ligand of Fas, exists in two types. The membrane-bound FasL is a 40-kDa type II transmembrane protein with a TNF homology domain (THD) that binds to Fas, whereas the soluble FasL (sFasL) is a 26-kDa protein fragment lacking the transmembrane and cytosolic domains [28]. Compared to Fas, FsaL is mainly expressed in lymphocytes, macrophages, and NK cells [29].

Mechanistically, upon binding of FasL to Fas, the adapter molecule Fas-associated death domain (FADD) is recruited to aggregation, and then FADD interacts with procaspase-8 by the death effector domain (DED) so as to form the death-inducing signaling complex (DISC). DISC initiates the activation of the caspase cascade that contributes to DNA fragmentation and cell-programmed death [29]. However, some Fas^+^ tumor cells can escape FasL-mediated apoptosis through downregulating Fas expression while increasing the expression of FasL or by serving as a novel Fas-resistant subpopulation due to immune-based selective pressure [30]. Thus, the role of this crosstalk in TME deserves further exploration.

#### 2.1.4. TRAIL-TRAIL-R

TNF-related apoptosis-inducing ligand (TRAIL, also known as TNFSF10 and APO2L) is a typical member of the TNF family, a 33 kDa acid type II transmembrane protein. Furthermore, the cysteine proteases can cleave the extracellular domain of TRAIL to produce a 20 kDa soluble TRAIL. TRAIL is expressed in various immune cells, including monocytes, DCs, and macrophages [31]. Its ligand, TRAIL-R, can be classified into two groups: the full-length intracellular DD-containing receptors TRAIL-R1 (DR4) and TRAIL-R2 (DR5), the alternative receptors TRAIL-R3 (DCR1), TRAIL-R4 (DCR2) and osteoprotegerin (OPG), both of which are distributed in various tissues [32]. The TRAIL-TRAIL-R axis is involved in spermatogenesis, the pathogenesis of pulmonary arterial hypertension (PAH), and tumor immune surveillance [31].

Upon TRAIL binding, the TRAIL-R manipulates apoptosis through a death-inducing signaling pathway, similar to Fas/FasL axis [32]. Moreover, targeting the TRAIL-TRAIL-R axis in TME has been confirmed to exert a profound effect on inhibiting tumor progression. For instance, TRAIL-R agonist-induced collapse of vascular integrity and apoptosis in tumors in a mouse fibrosarcoma model [33]. Although many TRAIL-based agents have already reached the clinical stage, further studies are needed to overcome TRAIL resistance in cancer therapy [32].

#### 2.1.5. SLAMF7-SLAMF7 Axis

Signaling lymphocytic activation molecule receptor family 7 (SLAMF7, also known as CRACC, CS1, CD319) is a member of the SLAM family. It is a single-pass transmembrane protein with an extracellular tyrosine-rich Ig-like domain and cytoplasmic tail, which carries an immune receptor tyrosine-based switch motif (ITSM) [34] and is usually expressed on hematopoietic cells [35]. SLAMF7 can mediate the development and function of lymphocytes cells and APCs, the generation of memory cells, the lytic viability of NK cells, the production of cytokines, the inhibition of MHC-independent cells, and the aggregation of platelet [36].

Significantly, as a homotypic receptor, SLAMF7 on macrophages can bind to SLAMF7 on cancer cells and act synergistically with macrophage-1 (MAC1) antigen, which is known to interact with two ITAM-containing receptors, FcRγ and DAP12 [37], and then induce cytoskeletal reorganization to phagocytosis tumor cells through SRC kinase, SYK, and BTK, rather than enhancing adhesion to targets cells [38]. Additionally, SLAMF7 knockout macrophages were defective in CD47 blockade-induced phagocytosis of mouse hematopoietic tumor cell lines [38]. This finding demonstrated that SLAM family receptors are required for tumor phagocytosis during the blockade of the SIRPα-CD47 axis. However, this view has been contradicted lately by Yuan et al., who presented that SLAMF7 expression on cancer cells is not required for phagocytosis [39]. Therefore, additional research is required to determine if SLAMF7 specifically interacts with the effects of CD47-targeting hematopoietic treatment.

#### 2.1.6. LRP1-CRT

Low-density lipoprotein receptor-related protein-1 (LRP1, also known as CD91 or α2M) is a 600-kDa transmembrane protein composed of a 515-kDa heavy (α) subunit and an 85-kDa light (β) subunit, the latter containing two YXXL motifs and two NPXY motifs, belonging to multifunctional LDL receptor (LDLR) gene family [40]. It is widely expressed on various cell surfaces, including smooth muscle cells [41], endothelial cells [42], and macrophages [43], and is involved in a variety of biological processes, such as inflammation, tissue remodeling, and clearance of extracellular molecules [44]. Calreticulin (CRT) acts as a recognition ligand of LRP expressed on the engulfing cell, which was discovered by Shyra J et al. in 2005 [45]. CRT is a member of the endoplasmic reticulum lectin chaperone protein family with a relative molecular mass of 46-kDa. It is highly species-conserved and mainly located in the endoplasmic reticulum (ER), which then binds to newly synthesized proteins to mediate their folding and glycosylation [46]. CRT is not only found on the surface of apoptotic cells but also in living cells. For example, the expression of CRT on cancer cells can mediate the uptake by APCs [47].

Increased expression of CRT on tumor cell membranes has been reported to bind to LRP1 on macrophages and initiate phagocytosis and immune response in target cells by triggering antigen presentation from APCs to T cells [45]. Furthermore, because CRT is also present in normal cells, the existence of the SIRPα-CD47 axis prevents the inadvertent uptake of phagocytes by these cells and, in turn, the anti-CD47 antibody-induced phagocytosis requires the interaction of target cell CRT with LRP on phagocytic cells [45]. Indeed, blockade or knockdown of CRT had comparable effects in suppressing the anti-CD47 antibody-mediated phagocytosis of cancer cells and abolishing their immunogenicity in mice [48].

#### 2.1.7. CLEC10A-sTn Axis

C-type lectin domain family 10 member A (CLEC10A, also known as CD301, MGL) was first identified back in 1996 [49]. It is a type II transmembrane receptor that is a member of the C-type lectin family and uses a common carbohydrate recognition domain to recognize glycan compounds in a Ca^2+^-dependent manner [50]. It is mainly expressed in intermediate monocytes, DCs, and macrophages [51]. Interestingly, CLEC10A expression is significantly lower in the majority of tumors than in normal tissues, which is linked to poor prognosis and cancer progression [52]. CLEC10A plays an active part in many biological processes, such as adaptive and innate immune responses [50]. Currently, several researchers have focused on the ability of CLEC10A to promote the anti-tumor activity of immune cells, and it has been reported that CLEC10A plays an indispensable role in increasing antigen-specific CD8^+^ T cell activation [53]. Beyond this, as a well-known epitope associated with human cancer, sialyl-Tn (sTn) Ag has been identified by C-type lectin expressed on macrophages as early as 16 years ago [49].

Naghmeh’s team verified transmembrane CLEC10A expressed on macrophages or dendritic cells interacts well with tumor-associated sTn (Neu5Acα2,6-Tn and Neu5Gcα2,6-Tn) in the micromolar range [54]. Unfortunately, the precise mechanism and implications of this interaction need to be clarified.

### 2.2. Rreceptor-Ligand Interaction in Pro-Tumor Immunity

#### 2.2.1. SIRPα-CD47 Axis

Signal regulatory protein alpha (SIRPα) is the first member of the SIRP family, which was identified in the 1990s, has an extracellular immunoglobulin-like (Ig-like) region and a cytoplasmic ITIM [55] and is mainly expressed on myeloid cells, including macrophages [56]. It is, therefore, an inhibitory receptor that has the ability to suppress intracellular signaling. The extracellular domain of CD47, which also contains five transmembrane domains and COOH-terminal splice variant tails [57], was initially discovered as a SIRP ligand in 1999 [58]. CD47 was initially discovered in ovarian cancer cells [59] but expressed in all cells and overexpressed in cancer cells as a “self-marker,” delivering an inhibitory “don’t eat me” signal by engaging in SIRPα, thereby avoiding phagocytosis by macrophages [60]. In addition, CD47 can participate in regulating cellular activities, including cell-cell fusion, cytokine production, migration, and activation [61,62]. 

When CD47 binds to SIRPα on the surface of macrophages, it leads to SIRPα cellular ITIM phosphorylation, which then recruits Src homology 2 (SH2) domain-containing protein tyrosine phosphatases 1 or 2 (SHP1/2) and activates them [63]. SHP1/2 dephosphorylates and inhibits myosin IIA, resulting in disruption of cytoskeleton rearrangement and phagocytosis activity, which in turn maintains homeostasis in vivo [64]. However, this pathway has also been reported to inhibit phagocytic synapses between macrophages and tumor cells in almost all tumor types, thereby promoting tumor immune escape [65]. Nowadays, substantial evidence supports that the checkpoint inhibitors of SIRPα-CD47 can suppress tumor growth by skewing tumor-associated macrophages toward the M1 phenotype from the M2 phenotype, which restores the phagocytic activities of macrophages [66]. Anyway, disruption of the SIRPα-CD47 axis can also activate adaptive immune responses, including amplifying the presentation of tumor-derived antigens to CD8^+^ T cell [67], or enhancing NK cell-modulated ADCC and complement-dependent cytotoxicity (CDC), thus alleviating suppression of the innate immune system [68]. However, because of its binding to CD47 on the surface of red blood cells (RBC), anti-CD47 treatment can lead to phagocytosis of RBC by macrophages and non-specific hemagglutination, which interferes with RBC antibody screening [69].

#### 2.2.2. PD1-PD-L1 Axis

Programmed cell death protein 1 (PD-1, also known as CD279) belongs to the B7 family and is a 55-kDa transmembrane protein first identified in 1992. It contains an extracellular IgV-like domain and a cytoplasmic tail with an ITIM domain [70]. PD-1 is constitutively expressed in immune cells, such as activated T cells, dendritic cells (DCs), and macrophages [71]. Moreover, it serves as an inhibitory receptor to control immune response. It has been shown that the level of PD-1^+^ TAMs increased during tumor progression in murine models, but the other subset of PD-1^–^ TAMs populations, which exhibit stronger phagocytic activity, decreased in this process [72]. Its ligand-programmed cell death protein ligand 1 (PD-L1, also known as CD274) is distributed not only on tumor cells and immune cells but also on other cells, such as vascular endothelial cells and pancreatic islet cells [73]. Moreover, in the 2000s, this checkpoint ligand was considered an important protein for immune evasion [74].

Mechanistically, when engaged with PD-L1, the ITIM domain of PD-1 expressed on macrophages will be activated and phosphorylated, which then mediates the activation of downstream pathways, such as SHP1 and SHP2, ultimately promoting anti-phagocytosis [72]. Nevertheless, the precise mechanism of the PD-1-PD-L1 axis in TAMs is poorly understood. Mediavilla et al. found that disruption of this axis leads to M1 polarization and activation of CD8^+^ T cells [72]. Additionally, PD-1 blockade increases tumor infiltration by anti-tumor macrophages while downregulating the number of pro-tumor macrophages, which then induces phagocyte-mediated anti-tumor immunity [75].

#### 2.2.3. LILRB1-MHC-I Axis

As a member of the leukocyte immunoglobulin-like receptor family, Leukocyte immunoglobulin-like receptor B1 (LILRB1) is distributed on the majority of immune cells [76] and osteoclasts [77], while paired immunoglobulin-like receptor B (PIR-B) is the only mouse receptor orthologous to the human LILRB family [78]. Both of them contain extracellular Ig-like regions and an intracellular ITIM motif that recruit SHP1 and SHP2, thus transducing an inhibitory signal [79]. Major histocompatibility complex I (MHCI) was considered the primary ligand of LILRB1 until 2003 [80], consisting of a heavy α-chain and β2-microglobulin (β2M). Moreover, it is species-specific, expressed on nucleated cells, and presents antigens to T cells [81].

In 2018, Barkal and his colleagues found that direct binding of LILRB1 to the β2M subunit of cancer cell MHC-I inhibits phagocytosis and disrupts immune surveillance by transducing intracellular inhibitory signals in TAMs [82]. However, interference with the MHC-I-LILRB1 axis through antibody blockade or genetic manipulation can drive tumor cells to be engulfed both in vitro and in vivo. The PIR-B-deficient macrophages showed an M1-like signature and increased proinflammatory cytokine release [83]. Furthermore, it is worth noting that MHC-I has the capacity to present specific peptides of tumor cells to T cells. Thus, it is optimized to target the β2-M or inhibit LILRB1 rather than disturb the binding of MHC-I-T cells to avoid affecting the cytotoxicity of CD8^+^T cells.

#### 2.2.4. MerTK-PROS1/GAS6-PtdSer Axis

MerTK is a component of the Tyro3-Axl-MerTK (TAM) family of receptor tyrosine kinases (RTKs) and contains two extracellular fibronectin type III (FNIII), two Ig-like domains, as well as a conserved kinase domain that characterized by the unusual KWIAIES sequence [84]. It is widely expressed or co-expressed by various cells and is involved in many physiological processes, such as tissue repair, clearance of apoptotic material, innate immune control, and platelet aggregation [85]. The two cognate molecules, protein S (PROS1) and growth arrest-specific protein 6 (GAS6), were identified as the ligands of TAM in 1995 [86]. PROS1 and GAS6 are produced by tumor cells and nonneoplastic cells in TME, which function as bridging molecules that are the physical link between MerTK and Phosphatidylserine (PtdSer) [87]. PtdSer, a kind of glycerophospholipid, is expressed on the inner leaflet of the phospholipid bilayer of normal cells and flips to the outer leaflet of the membrane on the apoptotic cells, providing an “eat me” signal to phagocytes [88].

MERTK on TAMs activated and started downstream signaling cascades after indirectly binding to PtdSer on dying tumor cells. This action led to the rearrangement of the actin cytoskeleton and the phagocytosis of macrophages, which strengthened the clearance of apoptotic tumor cells [89]. However, studies have shown that this interaction could induce macrophage polarization to M2 anti-inflammatory phenotype, secreting tissue repair-promoting cytokines, including IL-10 and TGF-β, ultimately facilitating immune escape [89]. The intervention of the MerTK-PROS1/GAS6-PtdSer axis may be a potent candidate for developing anti-tumor therapy. A study on murine MC38 colon cancer found that MerTK blockade induced an accumulation of apoptotic cells in cancers, the production of local type I IFN, and an increase in tumor immunogenicity, thus indicating the potential of cancer immunotherapy [90].

#### 2.2.5. Siglec15-sTn Axis

Sialic Acid-binding Immunoglobulin-like Lectin 15 (Siglec15), a member of the Siglec family of glycan-recognition proteins, is a vertebrate cell-surface receptor that can recognize sialylated glycans. Siglec15 is composed of two Ig-like domains, a transmembrane domain containing a lysine residue and a short intracellular tail. Moreover, it is linked to the signal adaptor molecule DNAX activation protein of 12 kDa (DAP12), which has an ITAM that activates immune cells [91]. The expression of Siglec15 was found to be restricted to myeloid cells, including macrophages and DCs in the spleen and lymph nodes [92]. In addition, it has been demonstrated that Siglec15 preferentially recognizes and binds to the sialyl-Tn antigen (sTn). sTn is a tumoral short O-glycan structure abnormally expressed in various tumors and associated with tumor metastasis [93].

Takamiya et al. first indicated that Siglec15 on TAMs can recognize sTn antigen on tumor cells and then transduces a signal to SYK through binding determinant Lys274 of DAP12 and enhance TGF-β secretion, which gradually facilitates tumor growth and metastasis. Additionally, THP-1 cells treated with SYK inhibitor or substitution of the Siglec-15 Lys274 to Ala suppress TGF-β secretion due to blocked interaction between Siglec15 and DAP12 [94].

#### 2.2.6. Siglec10-CD24 Axis

Sialic acid-binding immunoglobulin-like lectin 10 (Siglec10) is described as a member of the Ig-like lectin family, which can bind sialic acid and contains an intracellular ITIM domain [95]. In the meanwhile, it modulates cell-cell contacts and transmits inhibitory signals after being distributed to several immune cells, including macrophages and dendritic cells [96]. Sialoglycoprotein CD24 (also known as heat-stable antigen or small-cell lung cancer cluster-4 antigen) has been reported to connect with Siglec10 so as to mediate innate immunity [97]. CD24 is a highly glycosylated cell-surface protein and can be detected in various cell types, including cancer cells [98], exerting an inhibitory effect in autoimmune diseases, noxious inflammation, and cancer biology [99,100,101]. 

A single-cell RNA sequencing data has revealed that CD24 was overexpressed on a broad spectrum of tumors, and plenty of TAMs expressed a high level of Siglec10 [102]. Recently, many groups have discovered the anti-phagocytic signal of the Siglec10-CD24 axis in triple-negative breast cancer (TNBC) [102], mantle cell lymphoma (MCL) [103], and ovarian cancer [104]. The effect of Siglec10/CD24 takes place in a similar signaling pathway to PD1/PDL1 [105]. Furthermore, CD24 mAb treatment or CD24 deletion in tumor cell lines or mice can improve tumor cell phagocytosis, control tumor development, and lengthen life in vitro or in vivo by recovering the macrophages’ capacity [102].

#### 2.2.7. Ephrin-EphA4 Axis

Ephrin and its membrane-binding ligand Erythropoietin-producing hepatocyte kinase (Eph) are the largest receptor tyrosine kinases (RTKs) family. Ephrin and Eph both can be divided into two subclasses: EphrinA, EphrinB, and EphA, EphB, with an N-terminal extracellular molecular binding region, a transmembrane domain, and an intracellular kinase domain, the later one comprises of a juxta membrane (JM) domain, a tyrosine kinase domain (KD), sterile alpha motif (SAM) and PDZ binding motif (PDZBM) [106]. Their capacity to create signals with the property of bidirectional transduction, i.e., mediating both forward and backward signal cascades, was discovered to be expressed typically on a wide variety of cells [107]. This critical cell communication system plays widespread roles in cytoskeleton formation, cell adhesion, and movement, affecting neuronal development and skeletal stability [108].

Lu et al. demonstrated that EphA4 on CSCs can interact with TAMs by directly binding to Ephrin, subsequently stimulating Src and NF-κB and inducing a broad spectrum of cytokines secretion to sustain the stem cell state of the tumor [109]. However, it is worth noting that the role of the Ephrin-EphA4 axis in macrophages needs further study.

#### 2.2.8. EGF-EGFR Axis

Epidermal growth factor receptor (EGFR, also known as ErbB) is a membrane surface protein with tyrosine kinase activity, which contains an extracellular ligand-binding region, a single transmembrane region consisting of two repeated cysteine-rich regions, and an intracellular sequence containing tyrosine protein kinases and self-phosphorylation sites [110]. It is widely expressed in human epidermal and stromal cells but is highly expressed in various human cancer cells [111]. Epidermal growth factor (EGF), one of the seven ligands of EGFR, only binds to EGFR, whereas other EGFR ligands can bind to diverse members of the EGFR family [112]. The EGF-EGFR axis plays a crucial part in the physiological processes of cell growth, proliferation, and differentiation via RAS/RAF/ErK, PI3K/AKT, or Ral/c-Src/STAT pathway [113,114,115].

EGF-EGFR interaction was once thought to constitute a paracrine loop. However, it has been reported that EGFR can be activated through cell-cell interaction with its ligands [116,117]. Recently, Sevgi et al. used a multidisciplinary approach to investigate the interaction between BCC and macrophages and confirmed that this interaction is a juxtacrine loop. Moreover, cell-to-cell contact was required for EGF to stimulate EGFR on BCC [118]. Which fundamental mechanism controls this relationship, though, is still a matter of debate.

#### 2.2.9. SCF- c-Kit Axis

Stem cell factor receptor (c-Kit, also known as CD117), a member of the class III RTKs family, contains an extracellular (EC) ligand-binding domain, a single transmembrane (TM) region, and a juxtamembrane (JM) region [119]. C-Kit is a well-known proto-oncogene, and the encoded protein is expressed in several human tumor histotypes, including breast cancer [120], lung cancer [121], glioma [122], and leukemia [123]. The Stem Cell Factor (SCF), the counter receptor of c-Kit, is a type II homodimer of two four-helix bundles containing two distinct isoforms: soluble SCF (‘sSCF) and membrane-bound SCF (mSCF) [124]. 

SCF can stimulate c-Kit to mediate many cellular processes, such as proliferation, adhesion, differentiation, survival, and migration [125], via activating multiple downstream signaling pathways, such as Ras/Erk, JAK/STAT, and PI3K [126]. A recent study discovered that c-Kit, which is produced by ovarian CSC, interacts with cell-anchored, soluble SCF, which is expressed by myeloid cells such as macrophages or non-myeloid infiltrating cells, to promote the formation of tumors. Furthermore, this research also demonstrated SCF/c-Kit is a complex juxtacrine/paracrine circuit that is involved in enhancing ovarian CSC stemness properties. In vitro, the tyrosine kinase inhibitor imatinib can dampen the effect of activating the Akt pathway in c-Kit^+^ cells [127].

#### 2.2.10. CEACAM1-Metadherin Axis

Carcinoembryonic antigen-related cell adhesion molecule 1 (CEACAM1) belongs to the immunoglobulin superfamily, consisting of an extracellular domain, a transmembrane domain, and a cytoplasmic signaling domain with either a long (L) ITIM-containing domain or short (S) domain lack of ITIMs [128]. CEACAM1 is distributed on a variety of myeloid cells and lymphocytes. Furthermore, it plays a vital role in cell-to-cell adhesion, cell signaling pathway, and other complex biological processes such as inflammatory response and tumor progression [129,130]. A pluripotent oncogene known as Metadherin (MTDH, also known as AEG-1), which has been implicated in driving cancer metastasis, can directly interact with CEACAM1 [131]. Metadherin has also been discovered in other parts of cells, including the nucleus and endoplasmic reticulum [132].

More recently, Sally et al. identified a cell surface metadherin/CEACAM1-CCL3 positive feedback loop that can increase the formation of polyploid tumor cells and limit the efficacy of therapies in an isogenic tumor cell pair model of highly metastatic (HM) and non-metastatic (NM) tumor cells that resemble the spontaneous human ovarian cancer metastasis model. In addition, Metadherin on cancer cell surface communicated with CEACAM1 on macrophages to secrete C–C motif chemokine ligand 3 (CCL3), which promotes various tumor types of metastasis [131]. Since CEACAM1 has been reported could inhibit cell proliferation, and cytotoxicity of T cells and NK cells in melanoma, a new anti-CEACAM1 antibody (MRG1) was used to bind to the N domain of CEACAM1, restoring the susceptibility of melanoma cells to attacked by T cells [133].

#### 2.2.11. P-Selectin-PSGL-1 Axis

P-selectin sometimes referred to as SELP, is a cysteine-rich glycoprotein with a molecular mass of roughly 140,000 and is one of the essential members of the selectin family. It is expressed on the surface of activated platelets and endothelial cells [134]. Meanwhile, PSGL-1 is one of the ligands of P-selectin, ~120 kDa, with a homodimer structure linked by disulfide bonds, which is expressed chiefly on white cells, rarely on platelets, and strongly upregulated in M2-polarizing macrophages [135,136].

P-selectin interacts with PSGL-1 on macrophages, then accelerates leukocyte recruitment and migration to damaged endothelial cells. Moreover, it can mediate the mutual effect of leukocytes, endothelial cells, and platelets [137,138]. However, multiple myeloma (MM) gene expression profile data revealed that all investigated MM cell lines and primary MM cells showed PSGL-1, while all macrophages exhibited P-selectin. Meanwhile, P-selectin/PSGL-1 axis played a crucial part in macrophage-mediated MM drug resistance [139]. Therapeutically, PSGL-1 mAb can repolarize M2 macrophages to the M1-like phenotype and induce an inflammatory microenvironment, thus enhancing anti-tumor activity in myeloma cells and humanized mouse models [137,140].

#### 2.2.12. LSECtin-BTN3A3 Axis

Liver and lymph node sinusoidal endothelial cells lectin (LSECtin) is a transmembrane protein with a CRD that binds to extracellular ligands and belongs to the C-type lectin receptor family [95]. It was initially cloned by Liu et al. in 2004 and mainly expressed in hepatic sinusoidal endothelial cells, Kupffer cells, peripheral DCs, and macrophages [141]. LSECtin can combine with the Ebola virus, SARS-CoV, fibrillar virus, and hepatitis C virus [142,143,144]. Additionally, it can negatively regulate hepatic T-cell immunity [145]. Liu et al. also first identified BTN3A3 as the receptor for LSECtin [146]. BTN3A3 is one of the B7-related butyrophilins (BTN) family members, contains extracellular IgV and IgC domains, a transmembrane domain, and an intracellular B30.2 domain that show some structural features of the B7 family as well as distributed in diverse types of tumor cells [147]. The single-nucleotide polymorphisms (SNPs) in BTN3A3 demonstrated that it is related to the increase in susceptibility to ovarian [148].

According to research, BTN3A3 on TAMs might directly connect with LSECtin on BCC, which would afterward cause STAT3 to become phosphorylated and promote the expression of genes responsible for stemness. Based on this result, either macrophage-specific ablation of LSECtin or silencing of BTN3A3 in mice bearing human tumor xenografts can block the LSECtin-BTN3A3 axis, decreasing CSC frequency and tumor growth ultimately [146].

#### 2.2.13. Dectin1-Galectin9 Axis

Dendritic cell-associated C-type lectin1 (Dectin1) is a type II membrane protein with a molecular mass of 28 kDa that is encoded by CLEC7A, containing an ITAM on the intracellular tail. It is a member of the pattern-recognition receptors (PRR) C-type lectin family and is mostly expressed on myeloid cells [149]. Furthermore, it acts as a transmembrane signal receptor to mediate various functions of cells, including binding to eubacteria, inducing cytokine, and producing chemokine [150]. Subsequently, Galectin9 has been found to ligate Dectin1 in pancreatic ductal adenocarcinoma (PDA) [151]. Galectin9 belongs to the galactoside–binding family of lectins containing two domains, namely the N-terminal sugar recognition domain (N-CRD) and C-terminal sugar recognition region (C-CRD) and is expressed by lymphocytes and other cells [152]. Galectin9 plays a decisive role in physiological and pathological processes, such as disrupting the anti-cancer activity of cytotoxic lymphocytes [153] and activating anti-microbial innate immune responses in response to T cell suppression [154].

Especially, Galectin9 can activate Dectin1 on macrophages to promote pancreatic carcinoma and peritumoral immune tolerance, and the Dectin1–Galectin9 axis plays a decisive role in the education of T cells toward immunogenic or tolerogenic phenotypes in PDA. The immune-suppressive character of PDA-infiltrating T cells is reversed by Dectin1 blockade, which also results in anti-tumor immunity against PDA [151].

#### 2.2.14. α4-Integrin-VCAM-1 Axis

Integrin is a glycoproteins family that forms heterodimeric receptors for ECM molecules and includes one α subunit and one β subunit [155]. Moreover, it is distributed on many cell types of the hematopoietic lineage, such as T lymphocytes and macrophages, promoting adhesion to target cells [156]. Incidentally, α4-integrin belongs to this family. As a ligand of integrin and a member of the immunoglobulin superfamily, vascular cell adhesion molecule-1 (VCAM-1, also known as CD106) was first discovered in 1989 [157]. VCAM-1 predominantly binds to α4β1-integrin (also known as VLA-4) but only binds weakly to α4β7-integrin [158]. VCAM-1 is involved in numerous pathophysiological processes, including immunological disorders [159] and cardiovascular diseases [160]. However, several studies have also discovered aberrant VCAM-1 expression in various malignancies, including gastric, breast, and melanomas [161,162,163].

The binding of α4-integrin and VCAM-1 plays a major part in regulating leukocyte adhesion and transendothelial migration during inflammation [164]. VCAM-1-expressing tumor cells attract, ligate and activate α4-integrin-expressing osteoclast precursor cells to establish bone metastasis sites in breast cancer with bone metastasis. Furthermore, previous research also reported that VCAM-1 tethers metastasis-associated macrophages (MAMs) to cancer cells via α4 integrin and transduces a survival signal, which can protect cancer cells from apoptosis [165]. Meanwhile, this protumor function of VCAM-1 can be eliminated by the α4-integrin blockade and delay cancer bone metastasis [165,166].

#### 2.2.15. MAC1-CD90 Axis

Macrophage Antigen-1 (MAC1) is a heterodimeric complement receptor consisting of CD11b (α subunit) and CD18 (β subunit) integrins, which is expressed on different leukocyte subsets, including macrophages [167]. MAC1 is a key in triggering phagocytosis of complement fragment C3bi-opsonized particles [168], such as pathogens and apoptotic cells, in addition to promoting myeloid cell adhesion and migration [37,169]. CD90 (also known as Thy-1) is a glycosyl phosphatidyl inositol (GPI)-anchored cell surface protein with a molecular weight of 25~37 kDa, belonging to the IgSF [170]. Additionally, CD90 has been revealed to be a novel ligand for MAC1 by Wetzel and his colleagues [171]. CD90 is now known to be expressed in both cancer cells and normal tissue cells, such as endothelial cells, according to a recent report [172]. More importantly, CD90 can mediate cell adhesion and serves as a marker of cancer stem cells (CSC) [173].

Along with these, it has been shown that MAC1/CD90 can mediate the interaction between breast cancer stem cells (BCSCs) and TAMs in a contact-dependent way. However, CD90 provides the physical connection required for other cell-surface receptors, such as Ephrin/EphA4 juxtacrine signaling, since it lacks an intracellular domain [109]. Some studies revealed that knockdown or interference of CD90 by shRNA or miR-125a/b in TAMs, respectively, will result in suppression of cell proliferation and stem cell properties, the twofold to a threefold reduction in cytokine mRNA, finally avoiding tumor development [109,174].

#### 2.2.16. MAC1-ICAM-1 Axis

Intercellular adhesion molecule-1 (ICAM-1, also known as CD54) is a 90 kDa single-strand glycoprotein that belongs to the IgSF [175]. It is mainly distributed on the surface of endothelial cells, monocytes, lymphocytes, and cancer cells facilitating not just cell-cell adhesion and recognition but also the migration of immune cells such as leukocytes [176]. ICAM-1, thus, plays a crucial part in physiological processes such as cell signal transmission and activation, cell tissue development and differentiation, immunological response [177,178], and pathological processes, for example, tumor metastasis [179].

The previous study has shown that ICAM-1 is another ligand of MAC-1. According to the earlier study, MAC-1 on neutrophils binds to ICAM-1-expressing cells to mediate stable adhesion [180]. Additionally, it has been reported that ICAM-1 on TAMs could bind CD18 on Myeloma cells to regulate drug resistance [139], and this contact-dependent interaction tended to expand M2 infiltration and release cells from tumor cell aggregates [181]. The ICAM-1/CD18 axis in TME can form a positive autocrine feedback loop in ovarian cancer models, thereby promoting tumor cell proliferation, migration, spheroid formation, and peritoneal implantation. Subsequently, antibody blockade of ICAM-1 in macrophages blunted spheroid formation and ovarian cancer progression in these models [182].

## 3. Immune Checkpoint Receptor-Ligand Interaction Independent of Cell-Cell Contact

In addition to cell-cell contact, tumor cells can release plenty of molecules with associated receptors on macrophages (Figure 1 and Figure 2), such as CSF-1, Chemokines, and IL-10, resulting in the reprogramming of macrophages.

### 3.1. TLRs

Toll-like receptors (TLRs) are the best-characterized PRRs that control innate immunity. TLRs on macrophages are capable of detecting a wide range of molecules, including damage-associated molecular patterns (DAMP) generated from tumors, viruses, lipopolysaccharide (LPS), and bacterial nucleic acids, resulting in the activation of multiple signaling. These activated signal pathways can skew macrophages toward the M1-like phenotype, increase the secretion of proinflammation cytokines such as TNF-α and IFN-α, and upregulate the recruitment and activation of T cells to limit tumor progression [183]. Thus, targeting TLRs of TAMs may be a promising anti-tumor therapy. The majority of investigations, which use TLR agonists for facilitating immune responses against tumors, have focused on TLR3 [184], TLR4 [185], TLR7/8 [186], and TLR9 [187]. However, tumor-associated DAMP can also bind to TLRs expressed on macrophages to promote chemoresistance and metastasis [188]. Therefore, more research must be conducted to identify the precise contact mechanism between tumor cells and TAMs.

### 3.2. CSF-1R

Colony-stimulating factor-1 receptor (CSF-1R), restrictedly expressed on macrophages and frontier monocytes, is a major growth and differentiation factor belonging to the tyrosine kinase family. The CSF-1/CSF-1R axis is essential for monocyte recruitment, survival, and polarization [189]. In order to maintain an immunosuppressive environment, CSF-1 (also known as M-CSF) may be abundantly secreted from a variety of tumors and regulate the function of monocytes [190]. CSF-1R inhibitors can reverse the chemoresistance of breast cancer cell lines, increase the antigen presentation capacity of macrophages to enhance T cell responses, and reduce M2-like macrophage infiltration [191,192]. Numerous CSF-1R inhibitors and antibodies have been created and evaluated in clinical settings [193].

### 3.3. Chemokine Receptor

The chemokine receptor and ligand crosstalk between TAMs and tumor cells, such as C-C motif chemokine receptor 2 (CCR2)/C-C motif chemokine ligand 2 (CCL2), CCR5/CCL5, C-X-C motif chemokine 1(CXCR1)/CXCR2/CXCL8 (also known as IL-8), CCR4/CXCL12 (also known as SDF-1), CCR6/CCL20 (also known as MIP-3α), plays a crucial role in recruiting monocytes to infiltrate tumors, promoting macrophage polarization toward the M2-like phenotype and protecting tumor cells from chemotherapy-induced apoptosis [194,195]. Thus, it may be a promising anti-tumor strategy to target the chemokine receptor/ligand axis. Additionally, it has been demonstrated in several tumor types that blocking this macrophage signaling using inhibitors or antibodies might prevent the recruitment of M2-like TAMs, increase the functionality of tumor-infiltrating CD8^+^ T cells, and promote the response to immunotherapy [196].

### 3.4. Interleukin Receptor

The interaction between tumor-derived interleukins (ILs) and macrophages develop a network that regulates the efficacy of immunotherapy against cancer [197]. The two members of the tumor-derived IL family that have received the most research attention are IL-1 and IL-10, both of which are crucial in the development of tumors. As an immunosuppressive cytokine, IL-1 can bind to IL-1R on macrophages and recruit the signaling adaptor MyD88 that activates NF-κB, which maintains the expression of immunosuppressive genes in macrophages and the significant promotion of tumors in mouse melanoma models [198]. In colorectal cancer, IL-10 generated a similar immunosuppressive impact through the CaKMII-ERK1/2-STAT3 pathway [199]. Clinical study has shown that antibody treatments that target IL-1 or IL-10 are successful therapeutically [200].

### 3.5. ATP/Adenosine Receptor

The ectonucleotidases CD39 and CD73 are expressed in various immune cells and non-immune cells, including macrophages and tumor cells. Extracellular ATP (eATP), which is produced by stressed or dying tumor cells, may be bounded by CD39 in TME, which then dephosphorylates eATP to AMP. Sequentially, CD73 converts AMP to adenosine, which dampens the effect on the innate and adaptive immune response via activating adenosine receptors (ARs) expressed on immune cells [201,202]. Additionally, multiple groups have found an increase in anti-tumor responses in CD39-deficient mice [203], CD73-deficient mice [204], and AR-deficient mice [205] experiments, making this axis become a potential therapeutic target in cancer immunotherapy.

### 3.6. C5aR

C5a, a complement anaphylatoxin, is released from various cells, including tumors and myeloid cells, functioning through binding to its receptors C5aR on myeloid innate immune cells, such as macrophages, DCs, and neutrophils [206]. Traditionally, C5a has been considered a proinflammatory factor and is involved in multiple ways to sustain the inflammatory response. For example, C5a can activate macrophages to release proinflammatory molecules and trigger an oxidative burst [206]. The function of C5a in TME has been found to be dose-dependent, however. In turn, the elevated levels of C5a prevent complement activation and are linked to tumor immune escape [207]. A study on murine breast cancer has revealed that C5aR–mediated alveolar macrophages could inhibit Th1 cells but increase Th2 cells with lower tumoricidal activity, which facilitates lung metastatic burden [208]. Therapeutically, a combined PD-1/C5a blockade significantly reduces tumor growth and metastasis in lung cancer [209]. C3a has also emerged as a potential tumor growth mediator, while research is lacking [206].

### 3.7. VEGFR

Vascular endothelial growth factor (VEGF), one member of the growth factors family, can bind to VEGFR expressed on earliest hematopoietic progenitors and played a crucial role in the TME [210]. For instance, the VEGF/VEGFR axis may prevent immune cells from differentiating and functioning properly, hence promoting the metastasis of neoplasms [211]. Nevertheless, although known TAMs can be attracted by VEGF secreted from tumors into the tumor site, the precise mechanism of the VEGF/VEGFR axis between tumors and TAMs is unclear [210]. In preclinical glioblastoma models, it has been demonstrated that simultaneous suppression of angiopoietin-2 and VEGFR increases survival time via diverse mechanisms, including the normalization of vascular function, reduction in tumor burden, and modification of macrophage phenotype [212].

### 3.8. TGF-βR

Transforming growth factor β (TGF-β) is an immunosuppressive cytokine secreted by tumor cells and immune cells, which binds to TGF-R on a variety of cells and has a dual function in TME. TGF-β slows the metastasis of tumors in the first stages of their development. However, excessive TGF-β will prevent effector T cells from maturing and from producing cytokines [213]. Furthermore, it has been demonstrated that TGFβ derived from the tumor may skew macrophage to an M2-like phenotype, aiding in the development of an immunosuppressive TME [214]. By now, TGF-βR1 inhibitors have shown safety and tolerability in the clinical trial of advanced hepatocellular carcinoma [215].

### 3.9. SUCNR1

Succinate receptor (SUCNR1) is one kind of the G protein-coupled receptor (GPCR), highly distributed in mouse tissues, such as the kidney, liver, and spleen. After being stimulated by succinate, SUCNR1 may increase inositol trisphosphate (IP3) levels, calcium mobilization, extracellular signal-regulated kinase (ERK) activation, and the generation of nitric oxide (NO) and prostaglandin E2 (PGE2) in the human embryonic kidney (HEK) 293 cells [216]. Recently, Wu et al. have found that tumor-derived succinate could trigger SUCNR1-PI3K-hypoxia-inducible factor 1 alpha (HIF-1 alpha) axis to polarize macrophages into anti-inflammatory phenotype, which accelerates tumor progression in vitro and in vivo studies [217]. Targeting the succinate/SUCNR1 axis between tumor cells and TAMs may be a prospective anti-tumor method in the future.

## 4. Conclusions

This review summarizes the immune checkpoint and other receptor-ligand interaction between macrophages and tumor cells, which plays an indispensable role in anti-tumor immunity or pro-tumor immunity, such as SIRPα-CD47, PD1-PD-L1, FcγR-Antibody-Antigen, CSF-1/CSF-1R axis. In TME, tumor cells can suppress the growth, survival, antigen-presenting, and phagocytic ability of macrophages by governing multiple inhibitory and stimulatory signals. Therefore, the discovery and target of immune checkpoints are essential to restore macrophage anti-tumor functions. Meanwhile, this TAMs-targeted approach seems promising from a clinical perspective (Table 2). For example, some SIRPα-CD47 targeted clinical trials have entered phase III and manifested great effects in the treatment of cancer patients.

However, some problems remain unsolved: (1) Very little is known about the downstream crossover signal transmission pathway of IC on TAMs or tumor cells, although there has been a lot of bioinformatic analysis of IC expression profiles [218,219]. (2) Lack of insight into the regulatory network between TAMs and other immune cells under immunosuppressive conditions. (3) Due to immune or metastatic heterogeneity, different cancer types respond differently to the same immune checkpoint inhibitor (ICI). (4) Only a minority of macrophages-targeted drugs were approved by FDA (Table 3).

In conclusion, macrophage-oriented therapy is a potential new approach to fighting cancer without a shadow of a doubt. In addition, it is necessary to identify novel checkpoint receptor-ligand, elucidate the interaction mode between macrophages and tumor cells as well as other components in TME, and conduct more relevant clinical studies to design new strategies and combination regimens with improved specificity profiles to overcome resistance and eradicate cancer.

## Figures and Tables

**Table 1:** Immunomodulators that were approved by FDA.

Immunomodulators Type	Drug Name	Trade Name	Target	Representative Indications	Approved Date
**Checkpoint inhibitors**	Ipilimumab	Yervoy	CTLA-4	Melanoma	2011.03
	Pembrolizumab	Keytruda	PD-1/PD-L1	Melanoma	2014.09
	Nivolumab	Opdivo	PD-1/PD-L1	Esophageal carcinoma	2014.12
	Avelumab	Bavencio	PD-1/PD-L1	Merkel cell tumor	2017.03
	Durvalumab	Imfinzi	PD-1/PD-L1	Transitional cell carcinoma	2017.05
	Cemiplimab	Libtayo	PD-1/PD-L1	Cutaneous squamous cell carcinoma	2018.09
	Dostarlimab	Jemperli	PD-1	Endometrial carcinoma	2021.08
	Relatlimab	Opdualag (In combination with nivolumab)	LAG-3	Melanoma	2022.03
**Cytokines**	Interferon alfa-2a	NA	IFNAR1/2	Chronic myelogenous leukemia	1986.06 (Withdraw)
	Interferon alfa-2b	Intron A	IFNAR1/2	Hairy cell leukemia	1986.06
	rGM-CSF	Sargramostim	CSF2R	Leukemia	1991.03
	Aldesleukin	Proleukin	IL-2/IL-2R	Renal cell carcinoma	1992.05
	Peginterferon alfa-2b	Peg-Intron	IFNAR1	Melanoma	2011.03
**Small molecules**	Imiquimod	ALDARA	TLR7	Basal cell carcinoma	2004.07
	Pexidartinib	Turalio	KIT, CSF1R, and FLT3	Giant cell tumor of tendon sheath	2019.08
	Rintatolimod	Rintamod	TLR3	Renal cell carcinoma	2021.06

**Table 2:** Partial clinical trials of TAM-targeted immunotherapeutic strategies.

Strategy	TAM-Target	Intervention	Clinical Phase	Tumor Type	Combination	ClinicalTrials.gov Identifier
**TAM** **reprogramming**	CD40	RO7009789 (Selicrelumab)	I (completed)	Solid tumors	Atezolizumab (anti-PD-L1 mAb)	NCT02304393
		APX005M (Sotigalimab)	II (ongoing)	Unresectable and metastatic melanoma	NA	NCT04337931
		ADC-1013 (Mitazalimab)	I (completed)	Solid tumors	NA	NCT02379741
	TLR7	Imiquimod	I/II (completed)	Breast cancer	Radiation, Cyclophosphamide	NCT01421017
	TLR8	VTX-2337	I (completed)	Epithelial ovarian cancer, Fallopian tube cancer	Pegylated liposomal doxorubicin	NCT01666444
	TLR9	SD-101	I/II (completed)	Lymphoma	Epacadostat, Radiation	NCT03322384
	CSF-1R	Cabiralizumab	I (completed)	Advanced solid tumors	Nivolumab (anti-PD-1 mAb)	NCT02526017
		FPA008	I/II (completed)	Tenosynovial giant cell tumors	NA	NCT02471716
**Macrophage-mediated apoptosis**	TRAIL-R	Mapatumumab	I/II (completed)	Advanced hepatocellular carcinoma	Sorafenib	NCT01258608
			II (completed)	Advanced hepatocellular carcinoma	Bortezomib	NCT00315757
			II (completed)	NSCLC *	Paclitaxel, Carboplatin	NCT00583830
**Macrophage-mediated phagocytosis**	SLAMF7	Elotuzumab	II (completed)	MM *	Lenalidomide	NCT03411031
			III (completed)	Lymphoma, MM	Lenalidmide, Deamthasone	NCT01239797
			III (completed)	MM	Nivolumab (anti-PD-1 mAb), Pomalidmide, Deamthasone	NCT02726581
	CD47	Hu5F9-G4 (Magrolimab)	III (ongoing)	Acute Myeloid Leukemia	Venetoclax, Azacitidine	NCT05079230
		CC-90002	I (completed)	Hematologic neoplasms	Rituximab (anti-CD20 mAb)	NCT02367196
	SIRPα	TTI-621	I (ongoing)	Hematologic malignancies, Solid tumor	Rituximab (anti-CD20 mAb), Nivolumab (anti-PD-1 mAb)	NCT02663518
	LILRB1	AGEN1571	I (ongoing)	Advanced solid tumors	Balstilimab (anti-PD-1 mAb), Botensilimab (anti-CTLA-4 mAb)	NCT05377528
	PtdSer	Bavituximab	II (completed)	Metastatic oancreatic cancer	Gemcitabine	NCT01272791
			II (completed)	Non-squamous NSCLC	Paclitaxel/Carboplatin	NCT01160601
	MerTk	MRX-2843	I (ongoing)	NSCLC	Osimertinib	NCT04762199
	Siglec10	alemtuzumab	III (completed)	B cell chronic lymphocytic leukemia	NA	NCT00046683
			III (completed)	T-cell lymphoma	CHOP14 chemotherapy	NCT00646854
**I** **nhibiting the immuno-suppressive activity**	PD-1	SHR-121 (Camrelizumab)	III (completed)	Non-squmous NSCLC	Carboplatin, Pemetrexed	NCT03134872
	PD-L1	Atezolizumab	II (completed)	Advanced solid tumors	NA	NCT02458638
			IV (completed)	NSCLC	NA	NCT03285763
	Siglec15	NC318	I/II (ongoing)	Advanced or metastatic solid tumors	NA	NCT03665285
			II (ongoing)	Advanced NSCLC	Pembrolizumab (anti-PD-1 mAb)	NCT04699123
	EGFR	Erlotinib	IV (completed)	Non-squmous NSCLC	NA	NCT01609543
	Galectin9	LYT-200	I/II (ongoing)	Metastatic solid tumors	Chemotherapy, anti PD-1 inhibitor	NCT04666688
	CD39	JS019	I (ongoing)	Advanced solid tumors, Lymphomas	NA	NCT05374226
	CD73	AK119	I (ongoing)	Advanced or metastatic solid tumors	Cadonilimab (anti-PD-1/CTLA-4 mAb)	NCT04572152
	AR2A	Ciforadenant	I (completed)	Renal cell cancer, Metastatic castration resistant prostate cacer	Atezolizumab (anti-PD-L1 mAb)	NCT02655822
	C5aR	TJ210001	I (ongoing)	Advanced solid tumor	NA	NCT04947033
			I (ongoing)	Relapsed or refractory advanced solid tumors	NA	NCT04678921
	TGF-βR	LY2157299	II (completed)	Hepatocelllar carcinoma	Sorafenib	NCT02178358
		AVID200	I (ongoing)	Hepatocelllar carcinoma	NA	NCT01025206
**TAMs recruitment**	ICAM-1	BI-505	I (completed)	MM	NA	NCT01025206
	CCR2/CCR5	BMS-813160	II (ongoing)	NSCLC, Hepatocellular Carcinoma	Nivolumab (anti-PD-1 mAb), BMS-986253 (anti-IL-8 mAb)	NCT04123379
	CCR2	MLN1202	II (completed)	Metastatic cancer	NA	NCT01015560
	CXCR4	BL-8040	II (completed)	Acute myloid leukemia	Cytarabine	NCT01838395
	IL-1	Anakinra	II (completed)	Multiple myeloma, Plasma cell neoplasm	Dexamethaso-ne acetate	NCT00635154
			I (completed)	Metastatic/Refractory cancer	Denosumab, Everolimus	NCT01624766
	VEGFR	HMPL-013 (Fruquintinib)	III (ongoing)	Metastatic colorectal cancer	NA	NCT04322539
		Avastin (Bevacizumab)	IV (completed)	Non-squmous NSCLC	Platinum-based chemotherapy	NCT00451906

* MM, multiple myeloma; NSCLC, non-small cell lung cancer.

**Table 3:** Macrophages-targeted antibodies that were approved by FDA.

Target	Drug Name	Trade Name	Representative Indications	Approved Date
**VEGF/VEGFR**	Bevacizumab	Avastin	Colorectal cancer	2004.02
	Ramucirumab	Cyramza	Stomach cancer	2014.04
**EGFR**	Cetuximab	Erbitux	Colorectal cancer	2004.02
	Panitumumab	Vectibix	Colorectal cancer	2006.09
	Necitumumab	Portrazza	NSCLC *	2015.11
**CD38**	Daratumumab	Darzalex	MM *	2015.11
	Isatuximab	Sarclisa	MM	2020.03
**SLAMF7**	Elotuzumab	Empliciti	MM	2015.11

* MM, multiple myeloma; NSCLC, non-small cell lung cancer.
